# [Corrigendum] BMP‑6 inhibits the metastasis of MDA‑MB‑231 breast cancer cells by regulating MMP‑1 expression

**DOI:** 10.3892/or.2024.8822

**Published:** 2024-10-09

**Authors:** Fen Hu, Yunfeng Zhang, Mi Li, Lina Zhao, Jing Chen, Shuang Yang, Xiujun Zhang

Oncol Rep 35: 1823–1830, 2016; DOI: 10.3892/or.2015.4540

Subsequently to the publication of the above paper, an interested reader drew to the authors' attention that the pair of data panels shown for the invasion experiments in Fig. 2D on p. 1826 were strikingly similar to the ‘Control’ data panels shown for the Transwell assay experiments in Fig. 5C on p. 1829.

After having re-examined their original data files, the authors realized that Fig. 5C had been inadvertently assembled incorrectly. The revised version of Fig. 5, now featuring the correct data for the ‘231-control/Control’ and ‘231-BMP-6/Control’ experiments in Fig. 5C, is shown below. Note that the corrections made to this figure do not affect the overall conclusions reported in the paper. The authors are grateful to the Editor of *Oncology Reports* for allowing them the opportunity to publish this Corrigendum, and apologize to the readership for any inconvenience caused.

## Figures and Tables

**Figure 5.**
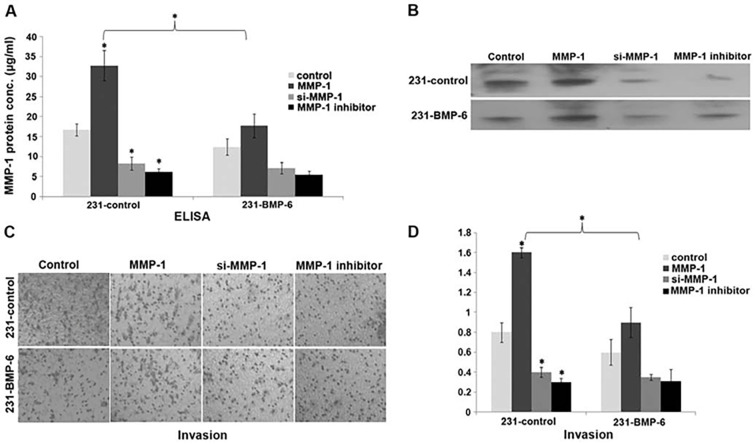
MMP-1 mediates the inhibition of cell invasion by BMP-6. 231-control and 231-BMP-6 cells were transfected with the MMP-1 expression plasmid or the si-MMP-1 plasmid or treated with the MMP-1 inhibitor (2 µM). MMP-1 protein levels in conditioned media were detected by ELISA assays (A) and western blotting (B). (C) 231-control and 231-BMP-6 cells were transfected with the MMP-1 expression plasmid, the si-MMP-1 plasmid or treated with the MMP-1 inhibitor (2 µM) and applied to the upper chamber of a Transwell-device with an artificial basal membrane. After 16 h, the cells passing the Matrigel and membrane were dyed using 0.25% crystal violet. (D) The crystal violet dye was washed off using 33% acetic acid, and the absorbance was measured on a spectrophotometer at 570 nm. *p<0.05, unpaired Student's t-test, compared with the control alone. BMP-6, bone morphogenetic protein-6.

